# Evaluating Listening Performance for COVID-19 Detection by Clinicians and Machine Learning: Comparative Study

**DOI:** 10.2196/44804

**Published:** 2023-05-09

**Authors:** Jing Han, Marco Montagna, Andreas Grammenos, Tong Xia, Erika Bondareva, Chloë Siegele-Brown, Jagmohan Chauhan, Ting Dang, Dimitris Spathis, R Andres Floto, Pietro Cicuta, Cecilia Mascolo

**Affiliations:** 1 Department of Computer Science and Technology University of Cambridge Cambridge United Kingdom; 2 Department of Medicine Vita-Salute San Raffaele University Milan Italy; 3 Electronics and Computer Science University of Southampton Southampton United Kingdom; 4 Department of Medicine University of Cambridge Cambridge United Kingdom; 5 Department of Physics University of Cambridge Cambridge United Kingdom

**Keywords:** audio analysis, COVID-19 detection, deep learning, respiratory disease diagnosis, mobile health, detection, clinicians, machine learning, respiratory diagnosis, clinical decisions, respiratory

## Abstract

**Background:**

To date, performance comparisons between men and machines have been carried out in many health domains. Yet machine learning (ML) models and human performance comparisons in audio-based respiratory diagnosis remain largely unexplored.

**Objective:**

The primary objective of this study was to compare human clinicians and an ML model in predicting COVID-19 from respiratory sound recordings.

**Methods:**

In this study, we compared human clinicians and an ML model in predicting COVID-19 from respiratory sound recordings. Prediction performance on 24 audio samples (12 tested positive) made by 36 clinicians with experience in treating COVID-19 or other respiratory illnesses was compared with predictions made by an ML model trained on 1162 samples. Each sample consisted of voice, cough, and breathing sound recordings from 1 subject, and the length of each sample was around 20 seconds. We also investigated whether combining the predictions of the model and human experts could further enhance the performance in terms of both accuracy and confidence.

**Results:**

The ML model outperformed the clinicians, yielding a sensitivity of 0.75 and a specificity of 0.83, whereas the best performance achieved by the clinicians was 0.67 in terms of sensitivity and 0.75 in terms of specificity. Integrating the clinicians’ and the model’s predictions, however, could enhance performance further, achieving a sensitivity of 0.83 and a specificity of 0.92.

**Conclusions:**

Our findings suggest that the clinicians and the ML model could make better clinical decisions via a cooperative approach and achieve higher confidence in audio-based respiratory diagnosis.

## Introduction

### Background

Over the past decades, digital transformation has promoted innovation in health care and biotechnology, yielding devices and machine learning (ML) models as well as enhancing processes and user experiences [1,2]. For example, computer-aided decision support systems have been developed and implemented to assist clinicians in various areas, such as augmenting limited intraoperative information and recommending surgical steps to surgeons by analyzing surgical videos and images [3]; identifying possible malignant tumors in terms of their location, size, and shape to assist clinicians in cancer diagnostics [4]; and exploring the drug-repurposing opportunities for new diseases, including COVID-19, by uncovering correlations among drugs, proteins, and diseases [5]. Although in many other health domains, the performance of humans and machines has been extensively investigated, compared, or even integrated [6-9], it has been understudied in the domain of audio-based respiratory health. In this study, we for the first time compare predictions from an ML model with predictions made by clinicians in audio-based respiratory disease diagnosis.

### Prior Work

Auscultation, that is, using an acoustic stethoscope to listen to the internal sounds of the body (eg, heart and lungs) is a test performed by physicians during routine physical examinations to confirm or rule out various medical conditions [10-12]. For instance, auscultation of the respiratory system, one of the oldest diagnostic techniques since the days of Hippocrates, is rather effective in detecting respiratory problems, such as asthma, pneumonia, and chronic obstructive pulmonary disease. Early lung auscultation studies associated characteristics of adventitious lung sounds (eg, wheezes and crackles) with specific respiratory diseases [11,13,14]. Unfortunately, several reports indicate that mastering auscultation remains a major challenge, and the auscultation skill of medical trainees and clinical physicians at all levels is declining with time [15-17]. With the aim of assisting clinicians to improve the performance of auscultation, a diverse range of signal processing and ML approaches have been developed, recording and analyzing body sounds via computer-aided digital auscultation systems [18,19]. Moreover, in addition to internal sounds collected via an acoustic medical device (eg, stethoscope) during physical examinations, breathing sounds and voice sounds can be collected and stored even remotely on mobile devices (eg, via embedded microphones in smartphones) and then analyzed to detect respiratory conditions. Recent research has demonstrated a high level of interest and promise of artificial intelligence–driven machine listening to detect respiratory pathologies in patients with active asthma, chronic obstructive pulmonary disease, and recently, COVID-19 [20-22]. These approaches are normally convenient, safe, affordable, and easy to perform.

Despite the rise of many novel approaches, a number of questions remain unanswered, including (1) what is the average performance of clinicians on human respiratory sounds, as opposed to lung sounds from standard auscultation? (2) Are there unique acoustic features specific to certain respiratory pathologies that are detectable by clinicians from sounds collected using a phone? (3) How do clinicians perform in remote audio-based respiratory diagnostics tasks in terms of both the prediction accuracy and the corresponding confidence, when compared with an automatic ML-based predicting system trained on this type of data? (4) and whether we could get improved performance in designing a combined decision system by integrating the intelligence of clinicians and machines, as there is a possibility that the two could be leveraging different aspects of the sounds associated with the disease, in which case complementary knowledge could lead to performance enhancement. Multiple reports were published about performance comparisons of clinicians and machines in the analysis of medical imaging (eg, computerized tomography, functional magnetic resonance imaging, and x-rays) [23,24]. However, to the best of our knowledge, there are no similar performance comparisons between clinicians and machines in terms of respiratory sounds.

### Goal of This Study

In this work, we selected COVID-19 sounds as a specific respiratory illness case study. Following our development of an audio-based digital testing model for COVID-19 prediction [22], this work presents a comparison study between clinicians and our model. Specifically, we compared the performance of over 30 clinicians and our ML-based model to examine human sounds for COVID-19 prediction, based on respiratory sounds (including breathing, coughs, and voice sounds) collected via a smartphone microphone from participants tested negative and positive for COVID-19. This study aimed to look into the agreement level of predictions of an ML model with suspected diagnosis of experienced clinicians and to lay the groundwork for the implementation of digital auscultation for remote respiratory condition assessment and monitoring.

## Methods

### Setting

This study aims to present a comparison of prediction by an algorithm (ie, a supervised trained model) and a human (ie, doctors with experience in treating COVID-19 or other respiratory problems) for COVID-19 from respiratory sounds collected from smartphone microphones. The Standards for Reporting of Diagnostic Accuracy (STARD) reporting guideline was followed [25].

### Overview

#### COVID-19 Sound Listening Test for Doctors

In this COVID-19 sound listening test, each clinician (referred to as “the doctor” below) was asked to listen to a set of respiratory sound samples (each about 20 seconds), blinded to their COVID-19 test labels. Some of the doctors worked in COVID-19 wards, and some others were clinical trainees. Each doctor was given access to these samples and performed the listening test independently. After listening to each sample, each doctor was asked to annotate with a yes or no whether they thought the sample was from a COVID-19–positive participant or not and to provide an uncertainty score between 1 (very uncertain) and 10 (very certain), describing how confident they were in this judgement. In this study, there was no time restriction for the clinicians to perform the test.

#### The ML Model

In our previous study [22], we developed and validated an ML framework (referred to as “the model” below) for detecting COVID-19 solely based on respiratory sounds of participants, crowd-sourced via mobile apps or web applications. The model was based on a deep convolutional neural network structure VGGish, which is a pretrained network that learnt knowledge from a large-scale general audio data set. The model was then trained on a data set consisting of 1162 samples from 400 COVID-19–positive and 400 COVID-19–negative subjects. Particularly, these audio samples were preprocessed by removing the silence periods at the beginning and the end of the recording and then normalized. We used Adam optimizer to reduce the binary cross-entropy loss function on these training samples (more details are presented in a study by Han et al [22]). Once trained, the model takes one sample, consisting of 3 respiratory sounds (ie, breathing, coughs, and voice) from the same subject as input. The sample is processed through the model, and the model outputs a two-dimensional prediction, indicating the probability of the sample being from a positive- or negative-tested participant, respectively. The categorical prediction is determined as the class with a larger probability, and this probability can be deemed as the confidence of the model for the given sample.

### Study Samples

In our previous study, audio data collection and research on audio-based COVID-19 prediction was performed [26,27]. In this study, we selected a subcohort from the original data collection, consisting of audio samples from COVID-19–positive and COVID-19–negative participants. Each sample includes a cough recording, a breathing recording, and a voice recording. We limited the number of samples to 24, to limit the burden of each doctor we involved, as it took them at least 0.5 hour to finish the listening test. The samples were randomly selected but needed to meet three criteria, as follows: (1) balancing the ratio of positive and negative, balancing gender, as well as spanning a wide age range; (2) covering various clinical presentations, so to include both symptomatic positive-tested and asymptomatic positive-tested individuals; (3) including other conditions or confounding factors, such as patients with active asthma and heavy smokers. It is important to mention that these selected samples were not used in the training of our ML model and were selected without knowledge of the outcome prediction of our model in advance. Once selected, these audio recordings were the only materials provided to the model and the clinicians, that is, additional information, such as the gender, age, and medical history, were neither used in the model nor known by the clinicians before they listened to the audio recordings. A summary of the basic information for each sample, including age, gender, complained symptoms, COVID-19 test results, and medical history, is listed in Table 1.

**Table 1:** Basic information of 24 selected samples (samples were selected to cover different gender and age groups with varied medical histories. Presence of symptoms reported among these samples are also listed).

ID	Gender	Age (years)	Medical history	COVID-19 test result	Symptoms
1	Male	30-39	—^a^	Negative	—
2	Male	20-29	Asthma	Negative	Sore throat and runny nose
3	Female	20-29	Other long-term condition	Positive	Wet cough, shortness of breath, dizziness, and headache
4	Male	50-59	High blood pressure	Negative	—
5	Male	20-29	—	Positive	Dry cough, wet cough, shortness of breath, tightness in chest, and sore throat
6	Female	50-59	Asthma, diabetes, or other long-term condition	Negative	Wet cough, shortness of breath, headache, and sore throat
7	Male	60-69	High blood pressure and diabetes	Negative	—
8	Female	30-39	—	Positive	Dry cough and loss of small or taste
9	Male	30-39	Asthma	Positive	Wet cough, tightness in chest, and loss of small or taste
10	Female	30-39	—	Negative	Dry cough
11	Male	60-69	High blood pressure and cancer	Positive	Headache
12	Female	60-69	—	Negative	—
13	Female	20-29	—	Negative	—
14	Female	40-49	—	Negative	Shortness of breath, headache, sore throat, and runny nose
15	Female	50-59	—	Positive	Wet cough, headache, sore throat, and dizziness
16	Male	40-49	—	Positive	—
17	Female	50-59	Asthma	Positive	—
18	Female	40-49	High blood pressure	Positive	Dry cough, tightness in chest, muscle ache, and dizziness
19	Male	50-59	—	Positive	Fever and wet cough
20	Female	40-49	—	Negative	Headache
21	Male	30-39	—	Negative	Wet cough, shortness of breath, sore throat, runny nose, and dizziness
22	Male	40-49	High blood pressure	Negative	Dry cough, wet cough, tightness in chest, sore throat, and dizziness
23	Male	40-49	—	Positive	Dry cough and muscle ache
24	Female	50-59	Asthma	Positive	—

^a^Not applicable.

### Study Design

This study compared the performance of audio-based COVID-19 predictions made by clinicians and an ML model. To this aim, 36 clinicians (with experience in treating COVID-19 or other respiratory illnesses) from four different sites were recruited to complete the COVID-19 sound listening test. After listening to each respiratory sound sample, they noted down their answers in a provided spreadsheet. Similarly, the trained ML model took each of the given samples as the input and returned the corresponding output prediction. We then examined the performance of the clinicians and the model and performed an in-depth comparison between the two.

Furthermore, we examined the performance of a committee of multiple members within a team, given that it is possible to reach a better performance by leveraging the power of a team. To this end, we evaluated the scenarios when different numbers of clinicians sit on a committee and make predictions collectively. More specifically, for each respiratory sample (consisting of breathing, cough, and voice sounds), a final decision was reached by applying weighted majority voting, taking all clinicians’ votes and their corresponding uncertainty scores into account. Moreover, the final uncertainty score was also derived by taking the average uncertainty score of the clinicians whose vote was the same as the final decision. We are also interested in exploring if the ML model can aid the clinicians, especially when conventional screening or testing tools are not available (eg, during web-based consultations). To simulate this setting, the ML model was considered as an additional member of the committee, with its votes and uncertainty scores being taken into account for the final decision.

### Statistical Analysis

Performance is assessed via 3 measurements, including sensitivity, specificity, and accuracy. Sensitivity indicates the performance to identify positive samples correctly, while specificity shows the ability to identify negative ones correctly. Accuracy, on this balanced cohort, can be applied to average the performance in terms of sensitivity and specificity. We computed 95% CIs of the metrics via bootstrapping. We examined the interrater agreement levels of the clinicians using Fleiss kappa (between multiple clinicians) and Cohen Kappa (between 2 clinicians) [28].

### Ethical Considerations

The study was approved by the ethics committee of the Department of Computer Science at the University of Cambridge (ID 2000). The participants gave written informed consent to participate in this study. The study data are deidentified.

## Results

### Performance of a Single Doctor and the ML Model

We present the performance of all 36 clinicians as well as the performance of the ML model (Table 2). Results showed that the ML model outperformed every single clinician, yielding 0.79 (95% CI 0.62-0.92) accuracy, 0.75 (95% CI 0.46-1.0) sensitivity, and 0.83 (95% CI 0.58-1.0) specificity for COVID-19 detection. In this study, the performance was evaluated on 24 selected samples for the ML model as well as for each clinician, while these samples were chosen to be representative. The same ML model was evaluated on a testing set with 200 participants, achieving 0.65 (95% CI 0.58-0.72) of sensitivity and 0.69 (95% CI 0.62-0.76) of specificity [22]. The clinician who achieved the best performance, ID 36, a pulmonologist working in the COVID-19 outpatient clinic, predicted the presence of COVID-19 with 0.71 (95% CI 0.50-0.88) of accuracy, 0.67 (95% CI 0.36-0.92) of sensitivity, and 0.75 (95% CI 0.50-1.0) of specificity. In this study, a small sample size (24) results in a wide CI range in the performance.

The concordance among all raters indicated a slight agreement level, with a Fleiss κ value of 0.137. Moreover, the paired interrater agreement from every 2 respiratory specialists based on Cohen κ is between –0.543 and 1.0, and the mean of all Cohen κ values is 0.162, also representing a slight agreement level on average between paired raters.

**Figure 1:**
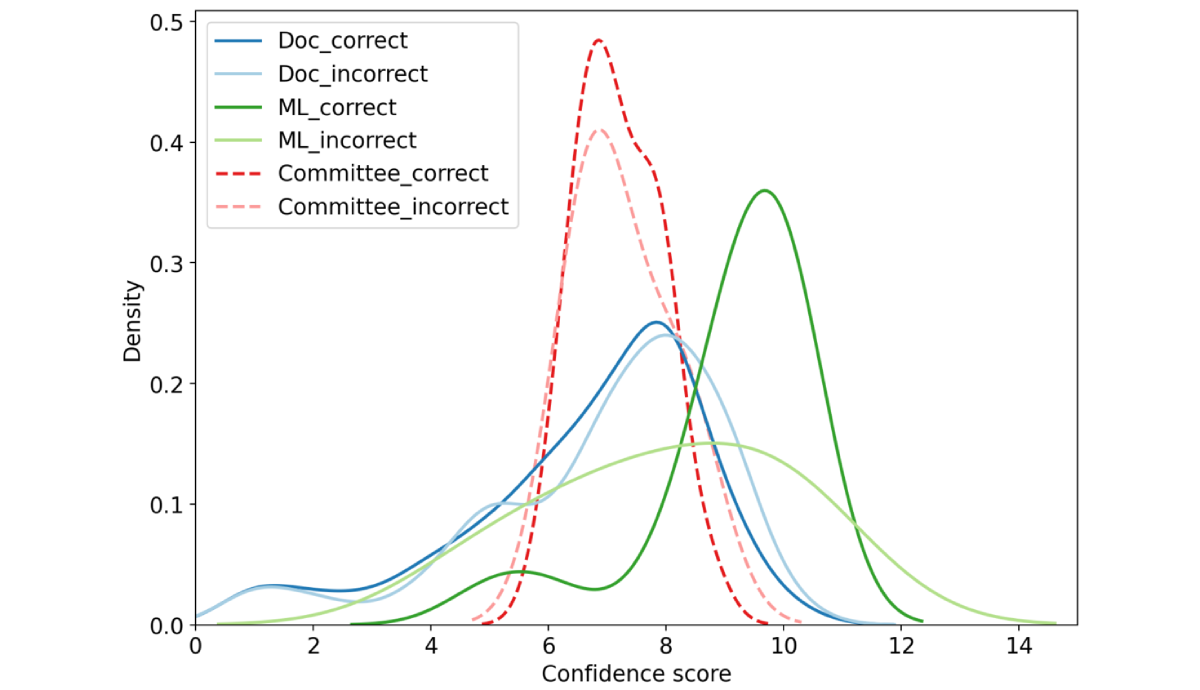
Confidence score kernel density estimate of 36 doctors and our model, for correct and incorrect predictions, respectively. Doc: doctor; ML: machine learning.

We further display the distribution of the uncertainty scores of all clinicians and the ML model for correct and incorrect predictions, respectively (Figure 1). Of note, with kernel density estimate, the resulting distribution estimation is smoothed and wider than the original range between 1 and 10. Distributions of the clinicians’ uncertainty scores for correct and incorrect samples (dark and light blue) are quite similar, covering the whole range and both centered at around 8. In contrast, uncertainty scores of the ML model are centered between 9 and 10, for both correct (dark green line) and incorrect (light green line) predictions. This implies an extremely high confidence level of the ML model for its predictions.

**Figure 2:**
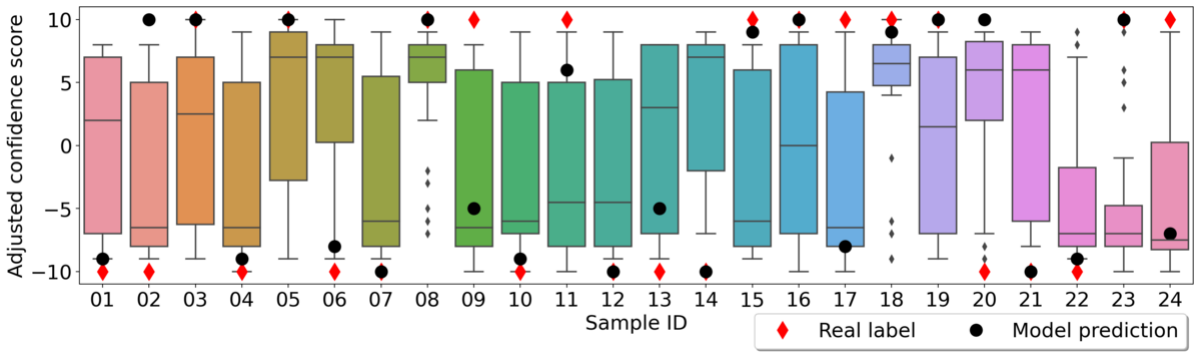
Confidence scores of all clinicians in box plot for each sample separately. Real labels and the model predictions are also displayed. The original confidence scores were adjusted to indicate the predictions or true labels (ie, if the prediction or label is COVID-19 positive, the confidence score is maintained; and if the prediction or label is COVID-19 negative, the original confidence score is multiplied by –1).

We listed the decisions of clinicians together with their uncertainty scores for each sample (Figure 2) in box plots. Specifically, for a COVID-19 negative detection, the uncertainty score was multiplied by –1; for a COVID-19 positive detection, the uncertainty score was kept unchanged. It can be noticed that although the opinions of the clinicians varied from each other in most of the samples, there were some samples where there was a high agreement among the clinicians with high certainties (eg, samples 08, 18, and 23).

**Table 2:** Performance comparison of 36 doctors and the machine learning (ML) model in terms of sensitivity, specificity, and accuracy, with 95% CIs. The best performance of 1 clinician and our ML model in terms of accuracy is italicized.

Doctor ID	Accuracy (95% CI)	Sensitivity (95% CI)	Specificity (95% CI)
1	0.29 (0.12-0.46)	0.42 (0.14-0.70)	0.17 (0.00-0.40)
2	0.54 (0.33-0.75)	0.50 (0.21-0.80)	0.58 (0.30-0.86)
3	0.25 (0.08-0.42)	0.33 (0.08-0.62)	0.17 (0.00-0.40)
4	0.33 (0.17-0.54)	0.25 (0.00-0.50)	0.42 (0.13-0.70)
5	0.46 (0.29-0.67)	0.50 (0.22-0.79)	0.42 (0.14-0.73)
6	0.42 (0.21-0.62)	0.33 (0.08-0.64)	0.50 (0.22-0.78)
7	0.46 (0.29-0.67)	0.50 (0.22-0.79)	0.42 (0.14-0.73)
8	0.62 (0.42-0.79)	0.75 (0.50-1.0)	0.50 (0.21-0.75)
9	0.25 (0.08-0.42)	0.33 (0.08-0.62)	0.17 (0.00-0.40)
10	0.62 (0.42-0.79)	0.75 (0.50-1.0)	0.50 (0.21-0.75)
11	0.33 (0.17-0.54)	0.25 (0.00-0.50)	0.42 (0.13-0.70)
12	0.42 (0.21-0.62)	0.33 (0.08-0.64)	0.50 (0.22-0.78)
13	0.54 (0.33-0.75)	0.50 (0.21-0.80)	0.58 (0.30-0.86)
14	0.42 (0.21-0.58)	0.25 (0.00-0.50)	0.58 (0.31-0.85)
15	0.54 (0.33-0.75)	0.83 (0.60-1.0)	0.25 (0.00-0.50)
16	0.50 (0.33-0.71)	0.33 (0.08-0.62)	0.67 (0.38-0.92)
17	0.50 (0.33-0.71)	0.33 (0.08-0.62)	0.67 (0.38-0.92)
18	0.62 (0.42-0.83)	0.58 (0.30-0.88)	0.67 (0.38-0.92)
19	0.58 (0.38-0.79)	0.50 (0.21-0.77)	0.67 (0.40-0.92)
20	0.62 (0.42-0.83)	0.58 (0.30-0.88)	0.67 (0.38-0.92)
21	0.58 (0.38-0.79)	0.50 (0.21-0.77)	0.67 (0.40-0.92)
22	0.50 (0.29-0.71)	0.75 (0.50-1.0)	0.25 (0.00-0.53)
23	0.54 (0.33-0.75)	0.42 (0.14-0.71)	0.67 (0.38-0.91)
24	0.50 (0.29-0.71)	0.50 (0.18-0.78)	0.50 (0.20-0.80)
25	0.62 (0.42-0.83)	0.50 (0.20-0.78)	0.75 (0.50-1.0)
26	0.54 (0.38-0.75)	0.33 (0.08-0.62)	0.75 (0.50-1.0)
27	0.50 (0.29-0.71)	0.50 (0.18-0.78)	0.50 (0.20-0.80)
28	0.46 (0.29-0.67)	0.42 (0.12-0.70)	0.50 (0.22-0.75)
29	0.29 (0.12-0.46)	0.42 (0.14-0.70)	0.17 (0.00-0.40)
30	0.62 (0.42-0.79)	0.75 (0.50-1.0)	0.50 (0.21-0.75)
31	0.58 (0.38-0.79)	0.42 (0.12-0.71)	0.75 (0.50-1.0)
32	0.58 (0.38-0.79)	0.58 (0.29-0.86)	0.58 (0.31-0.85)
33	0.50 (0.29-0.71)	0.33 (0.08-0.62)	0.67 (0.38-0.92)
34	0.50 (0.29-0.71)	0.33 (0.08-0.62)	0.67 (0.38-0.91)
35	0.62 (0.42-0.83)	0.58 (0.27-0.85)	0.67 (0.42-0.92)
36	*0.71 (0.50-0.88)*	*0.67 (0.36-0.92)*	*0.75 (0.50-1.0)*
ML model	*0.79 (0.62-0.92)*	*0.75 (0.46-1.0)*	*0.83 (0.58-1.0)*

### Performance of a Committee of Multiple Members

As shown in the previous section, the average performance of clinicians on the listening test is outperformed by the ML model. Though most of the clinicians had working experiences in a COVID-19 ward, they were not trained to detect COVID-19 based on respiratory sounds. In contrast, the ML model was developed on purpose to carry out audio-based COVID-19 prediction, training on 1162 samples from 800 participants (400 COVID-19–positive participants and 400 COVID-19–negative participants).

**Figure 3:**
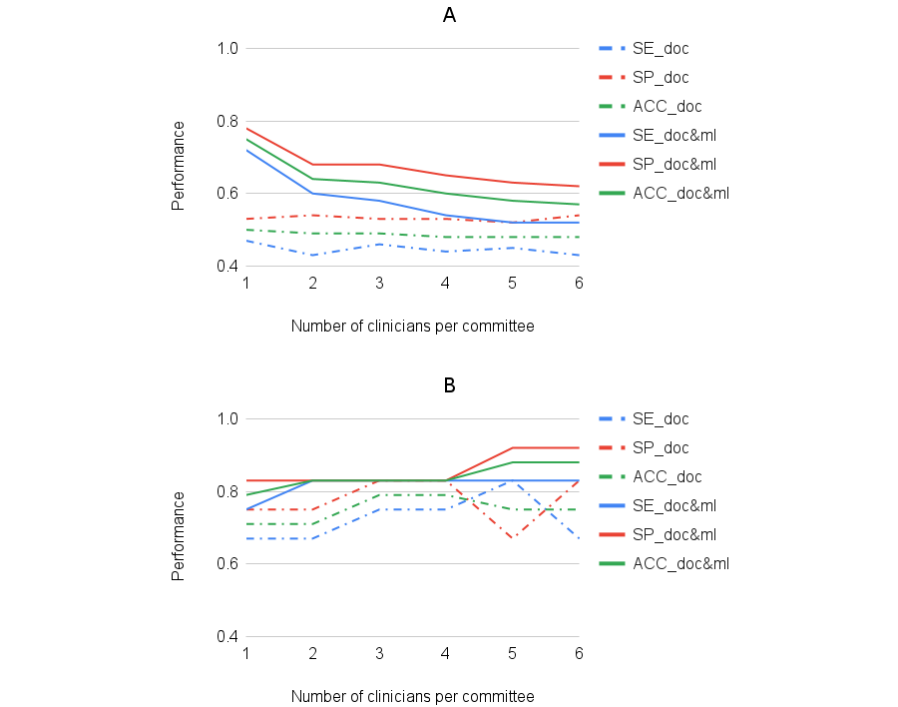
Performance of a committee of multiple members, in terms of sensitivity (SE), specificity (SP), and accuracy (ACC), respectively. (A) the average performance over 10000 times of committee selection. (B) The best performance that one committee with a certain number of members can obtain. Dashed lines are performance of the committee with doctors only, and solid lines are performance with the ML model as an additional member. Doc: doctor; ML: machine learning.

In this section, we reported the performance of a committee of multiple members. We presented the averaged performance of a committee with a varied number of clinicians (Figure 3A). Dashed lines are for performance in terms of accuracy, sensitivity, and specificity, respectively, between 1 and 6 (committee performance with more than 6 clinicians was not given here, as including more than 6 does not bring further performance increase). Further, we presented the best performance that could be achieved by a committee (Figure 3B). The results were based on random selections of committees over 10,000 times. It can be seen that the best committee with 3 or 4 clinicians obtained an accuracy of 0.79, sensitivity of 0.75, and specificity of 0.83. This was better than any single clinician and matched the performance of our ML model (Table 2). Of note, in this study, the best clinicians committee was selected based on their overall performance on the limited provided samples. However, in reality, such a committee might be selected based on the clinicians’ experience with the disease.

When decisions of the ML model were also considered in the committee, the performance of the adjusted committee is shown with solid lines (Figure 3). The figure shows that on average the ML model helped the most when a committee had a small number of clinicians, and the benefits declined as the number of clinicians increased. This is because, under the used voting strategy, the more human clinicians being involved, the less helpful the model was, as its opinion was diluted in the doctors’ collective. However, it is encouraging to observe that when the ML model was combined with the best clinicians committee (Figure 3B) with 5 or 6 clinicians, the performance improved, reaching an accuracy of 0.88, a sensitivity of 0.83, and a specificity of 0.92. The uncertainty score distribution of this best combination is also demonstrated (red lines in Figure 1), mainly centered around 7.

## Discussion

### Principal Results

In this work, we made a first step toward the integration of machine intelligence and human intelligence in the context of respiratory health. Specifically, we presented a detailed performance comparison of multiple clinicians and an ML model for audio-based COVID-19 detection from breathing, cough, and voice sounds. The ML model trained on thousands of samples outperformed the clinicians. However, it was overconfident when making wrong predictions. We provided insights on how, via proper cooperation, clinicians and the ML model could achieve a more accurate diagnosis, with a more appropriate confidence level. Furthermore, we showed the COVID-19 signatures in different sound types that helped clinicians make a prediction based on respiratory sounds only. To the best of our knowledge, this is the first reported performance comparison of a model with respiratory experts for detecting COVID-19 from audio.

The results have shown that the performance of a committee combining multiple doctors and the ML model outperformed the performance of the ML model alone as well as outperforming a committee of doctors without ML, indicating the possibility and potential benefits of integrating the predictions of clinicians and an ML model. Further, the derived uncertainty score distribution of the doctors-and-ML committee may imply that not only can the ML model help the clinicians committee reach a more accurate decision, but also clinicians help alleviate the over-confidence issue of the ML model.

**Figure 4:**
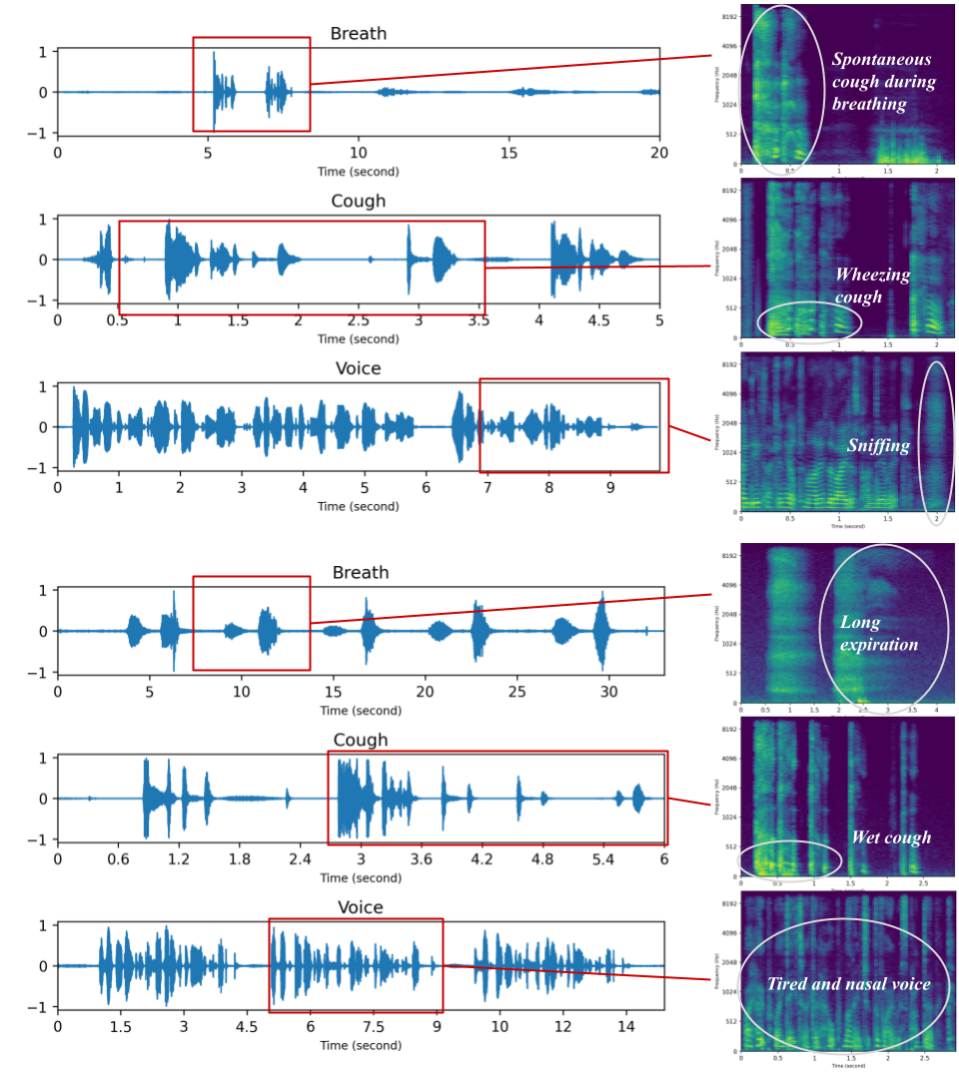
COVID-19 positive breath, cough, and voice recordings from 2 samples (08 and 18) and the reported cues from one of the clinicians.

We further invited the clinician with the best performance to list the reasons behind each decision. It should be noted that the ML model and this clinician reached an agreement of 0.328 in terms of Cohen Kappa score, indicating a fair consensus between the two. The clinician noted down several potential COVID-19 signatures from the audio, including nasal voice, wheezing cough, long expiration, tired voice, and strong coughs. we have shown 2 positive samples as examples in Figure 4. The clinician integrated the signatures from the 3 sound types and correctly diagnosed a positive COVID-19 case for both samples. This implies the complementarity and usefulness of the 3 respiratory sounds for audio-based COVID-19 screening.

When looking into mistakes of the ML model, the model made 5 mistakes (02, 09, 17, 20, 24), 4 of which were for participants with asthma (02, 09, 17, and 24; Figure 2). Moreover, 17 and 24 were samples from asymptomatic COVID-19–positive participants, whereas 02 was a sample from a COVID-19–negative participant who had respiratory symptoms, such as sore throat and runny nose, which also affect voice production. This was in line with the 2 of our previous findings [22], as follows: (1) for participants with asthma, the performance in terms of sensitivity declined to 0.33 (95% CI 0.07-0.64); and (2) the model was better in predicting symptomatic COVID-19 than asymptomatic COVID-19. Although the samples were selected to cover as many conditions as possible, the limited size of the sample hindered us to disentangle these confounders for a clearer view of the errors made by the ML model.

### Limitations

Several limitations to our study should be acknowledged. The samples used were crowd-sourced; therefore, the information collected, such as COVID-19 labels and the symptoms, were self-reported. Nevertheless, several publications have assessed the usefulness of our samples and obtained promising results [29-31].

Moreover, this is a relatively small study with 24 samples and 36 clinicians. Communicating with and gathering a statistically significant number of clinicians to perform the listening test was laborious. Aiming at promoting doctors’ engagement in developing artificial intelligence–driven assistive systems for health care, further investigation on such performance comparison and aggregation would be necessary, with more samples and clinicians involved.

In addition, all of the samples used in this study were in English language. Therefore, it is unclear whether and how different languages may affect the performance of the clinicians and the ML model, which was trained on English samples only, since language is a potential confounding factor for audio-based COVID-19 prediction [22]. In the future, we plan to study audio samples from Italian participants, which have the second largest number of samples in our collected data set.

Although only respiratory sounds collected from smartphones were considered in this study, in the future, it is also interesting to assess the ability of lung sounds collected via digital stethoscopes. Combining the knowledge of the 2 types of sounds might yield better prediction performance.

Finally, yet importantly, we considered the performance comparison between clinicians and the ML model on sounds only, as this was the main scope of our study. As the next step, it seems reasonable to consider additional information for clinicians or models to perform a more comprehensive and holistic analysis. Such information could include but is not limited to reported symptoms [32], body temperature [33], heart rate [34], and oxygen saturation level [35].

### Conclusions

In this study, we compared clinicians and an ML model in audio-based COVID-19 prediction and observed that the diagnostic accuracy of the model was superior to that of respiratory clinicians. The study also showed that the model and clinicians might be using complementary information, and by combining the predictions, it is possible to achieve a further increase in diagnostic accuracy. This study was the first study of its kind in comparing the performance of respiratory clinicians with an ML model and showed the value of a computer-aided decision support system in the transformation of respiratory care pathway. In light of the model’s ease of implementation, it can be applied to many scenarios, such as remote assessments, web-based consultations, and remote monitoring. Future work will focus on verifying such an ML-based system on other respiratory conditions and assessing its feasibility and acceptability in clinical practice.

## Abbreviations

ML: machine learning

## Data Availability

Anonymized data will be made available for academic research upon requests directed to covid-19-sounds@cl.cam.ac.uk. Academic institutions will need to sign a Data Transfer Agreement with the University of Cambridge to obtain the data. We have this agreement in place; please contact covid-19-sounds@cl.cam.ac.uk to obtain it. Once the document is signed, we will provide a download access to where the data is stored.
